# Association of MTHFR gene polymorphism C677T (rs1801133) studies with early primary knee osteoarthritis in a South Indian population: a hospital-based study

**DOI:** 10.4314/ahs.v22i1.41

**Published:** 2022-03

**Authors:** Subhadra Poornima, Swarnalatha Daram, Rama Krishna Devaki, Ramchander Merugu, Krishna Subramanyam

**Affiliations:** 1 Dept of Genetics and Molecular Medicine, Kamineni Life Sciences, Moula-Ali, Hyderabad, 500040; 2 Dept of Biochemistry, Kamineni Academy of Medical Sciences and Research centre, LB Nagar, Hyderabad, 500074; 3 Dept of Biochemistry, Mahatma Gandhi University, Nalgonda, 508254; 4 Dept of Orthopaedics, Yashoda Hospitals, Malakpet, Hyderabad,500036; 5 Dept of Genetics and Molecular Medicine, Kamineni Academy of Medical Sciences and Research Centre, LB Nagar, Hyderabad, 500078

**Keywords:** Polymorphism, MTHFR gene, Osteoarthritis, molecular analysis

## Abstract

Osteoarthritis (OA) is the most commonly occurring disease of middle and elderly population, which is characterized by focal loss of joint articular cartilage, osteophyte formation and sub chondral bone remodeling. Classical risk factors of OA include age, gender, weight, joint injury, trauma, however hereditary component is one of the main crucial factors. Several genome wide association studies and candidate gene approaches have identified genetic variants involved in the influence and association of OA. In the current study influence of Methylene tetra hydro folate reductase MTHFR C677T (rs1801133) gene with early primary knee OA was evaluated.

In this study 400 samples were included (200 cases & 200 controls). DNA was extracted & processed for PCR- RFLP evaluation and genotype analysis. Statistical analysis was performed & results indicated a lack of association between MTHFR gene polymorphism and early primary KOA. The stratification was done based on age & gender and also both. Individual's i.e females below the age of 40 years are more prone to the disease when compared with males. MTHFR gene polymorphism showed a lack of association with early primary knee osteoarthritis. To the best of our knowledge this is the first study from south India.

## Introduction

Osteoarthritis (OA) is one of the most common types of arthritis associated with musculoskeletal disorders, affecting an estimated 302 million people in the middle and elderly age groups globally^1^. There is a greater proportion of women in favor of OA than men which is predicted to be attributable to the aging^2^. The disease is caused by both environmental and genetic factors. However, the genetic risk factors can have an impact on whether or not the disease develops, how rapidly the disease progresses, and how severe the symptoms appear. It can have an effect on obesity as well as bone structure. There may be genetic factors that influence the disease's development as well as its likelihood of occurrence. Obesity and having a natural or ideal body weight are also risk factors^3^. The genetics of OA are complex and not completely understood. Several epidemiological studies have suggested a genetic contribution. The turnover is synergistic between a gene-activated mechanism complex involving injury and its response, body weight, muscle mass, bone, and cartilage structure^4^, ^5^. OA affects the knee, hands, hip, and spine, but has the greatest effect on the vertebrae. In the patient, it inflicts severe pain and disability, which means that OA has a tremendous effect on the population^6^. The prevalence of chronic knee pain over the past couple of decades has risen by nearly 65% and now affects about 25% of all adults^7^. Knee Osteoarthritis (KOA) is known for the degenerative joint disease is caused by cartilage and underlying bone breakdown^8^. Age, gender and ethnicity are the prime causes for the incidents of KOA. However, there are numerous sources for arthritis; the clinically most common knee in our population^9^. Around 30% of older people over the age of 45 have radiographic confirmation of KOA, with about half of those experiencing knee symptoms. Obese people have a higher lifetime risk of symptomatic KOA than non-obese subjects^10^.

Despite significant advances in molecular biology techniques, the precise mechanism of the disease remains unclear 11. Twin studies, analysis of segregation and linkage and studies of candidate genes have provided important knowledge on patterns of inheritance and the genome location of possible causative mutations in the human diseases^12^. It is apparent that genetic predisposition from genome wide associations studies (GWAS) influences OA susceptibility. Many loci are relevant to the creation of OA in established literature^13^. Candidate genes were investigated due to their critical roles in the pathogenesis of OA. Several candidate genes have been linked to the phenotypic manifestation of early KOA in various population-based studies. Among them, Methylene Tetra HydrofolateReductase (MTHFR; OMIM:607093) is a regulatory enzyme of folate and homocysteine metabolism, is one of the candidate genes for osteoarthritis growth^14^. MTHFR gene has been located on chromosome 1p36.22. A common C to T transition in the MTHFR gene at nucleotide 677 (C677T) results in the replacement of alanine by valine in the protein structure. This genetic polymorphism is found in exon 5 of the MTHFR gene, which corresponds to the protein's folate binding site. The existence of this polymorphism has been linked to increased MTHFR thermo lability and decreased specific activity. This mutation is thought to be the most common genetic cause of high homocysteine levels^15^–^17^. MTHFR is a good candidate gene for OA because this polymorphism has been linked to the inflammatory process. In the Indian population and with KOA disease, limited studies were documented and none of the studies were carried out with C677T polymorphism and KOA. Hence, the current study aims to investigate the genetic relation between C677T polymorphism in KOA patients diagnosed in South Indian population.

## Materials and methods

### Participants involved in this study

**Figure 1:**
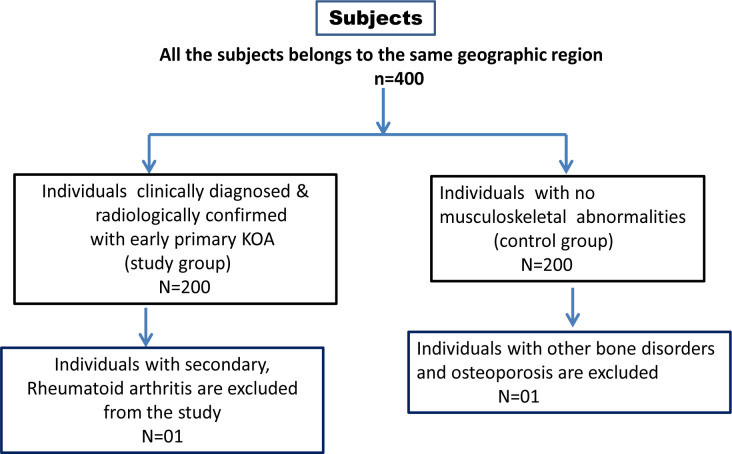
Selection criteria of the study subjects

This case-control study was implemented after the grant of ethical approval from institutional committee within the premises of Kamineni Hospitals, capital city of Telangana, India (KHL No. e374/13). Simultaneously, the participants who were recruited in this study has signed the consent form. In this study, based on inclusion and exclusion criteria, we have opted 200 KOA cases and 200 healthy controls (Figure-1). Individuals that have been clinically diagnosed with early primary KOA and have had radiological tests support their diagnosis. In terms of KL grading, all patient grades were considered as the inclusion criteria for KOA cases. The exclusion criteria of KOA cases are infectious, rheumatoid, and secondary osteoarthritis. The healthy controls are selected randomly, with a normal body mass index (BMI) and no personal and family history of any form of arthritis, and who have visited the master health check-up for routine tests. The control subjects with other diseases, family history, and abnormal BMI were excluded from this study. The age and gender matching KOA cases and controls were recruited. The mean age of the KOA cases were 44 years and 43 years was found to be the mean age of healthy controls involved in this study.

### Sample collection

3ml of peripheral blood was obtained from study participants in an EDTA vacutainer for DNA isolation and molecular analysis at the department of Genetics and Molecular Medicine, Kamineni Life Sciences, Moula -Ali, Hyderabad.

### Involvement of anthropmetric measurements and other details

Age and gender information was recorded for both cases and controls. Height was measured in centimeters, weight was measured in kilograms, and BMI was determined using WHO guidelines 18. KOA cases were allocated as per KL classification based on the nature of their symptoms and X-ray observations. Information on co-morbidities such as hypertension, type 2 diabetes, and thyroid dysfunction were obtained from both cases and controls. A family history of osteoarthritis was also obtained in the family pedigree.

### Molecular analysis

According to the previous research incorporated by our lab, the C677T polymorphism of the MTHFR gene was chosen from the candidate gene approach to test in our population^15^. DNA was extracted from both cases and controls using the same salting out technique used in our routine lab, and samples were stored at -20o C before further use^19^.

Initially, a 25-µl reaction containing 50-100ng of genomic DNA, 10pmoles of primers, 10X buffer, 2mM Mgcl2, 0.5mM dNTPs mix, and 5 units of Taq DNA polymerase (Ferments) is adjusted with distilled water for the final volume. PCR conditions for the reaction are as follows: initial denaturation (95°C-5mins), denaturation (95°C -30sec), annealing (65°C -45sec), extension (72°C -45sec), and final extension (72°C -7mins), with a hold at 4°C after 35 cycles^20^. The PCR amplification for MTHFR gene of C677T; specific primers were used, with an amplicon scale of 198 base pairs 15. Table-1 contains the details of C677T polymorphism. The restriction enzyme Hinf1 was used to perform RFLP to understand the nucleotide transition at position 677 that causes the amino acid to shift from Alanine to Valine. The digested products were then electrophoresed on a 2% ethidium bromide stained gel (Figure-2). Prior to genotyping study subjects, a few samples were validated with Sanger sequencing parallel to RFLP (Figure-3).

**Table 1:** Details of SNP evaluated in the study with early primary knee osteoarthritis and controls

S. No	SNPedia	Orientation Details
1	Gene	MTHFR
2	Reference sequence number	rs1801133
3	Condition	Early primary knee Osteoarthritis
4	Organism	Homo sapiens
5	Mutation	Non-synonymous single nucleotide polymorphism
6	Amnio acid substitution	C677T (Ala 677 Val)
7	Single Nucleotide Polymorphism	C-T
8	Exon position	Exon 5
9	Chromosome Region	Chromosome-1 p36.22
10	5′-3′ Primer sequence	TGAAGGAGAAGGTGTCTGCGGGA
11	3′-5′ Primer Sequence	GGACGGTGCGGTGAGAGTG
12	PCR Amplicon size	198bp
13	Restriction Enzyme	Hinf1
14	Substitution of Nucleotide band size	176bp
15	Digested amplicon size	CC: 198bp ; CT:198/176/282bp ; TT176/22bp
16	Genotype effect	CC: Normal or no risk CT: Increased risk TT: Increased risk

**Figure 2 A:**
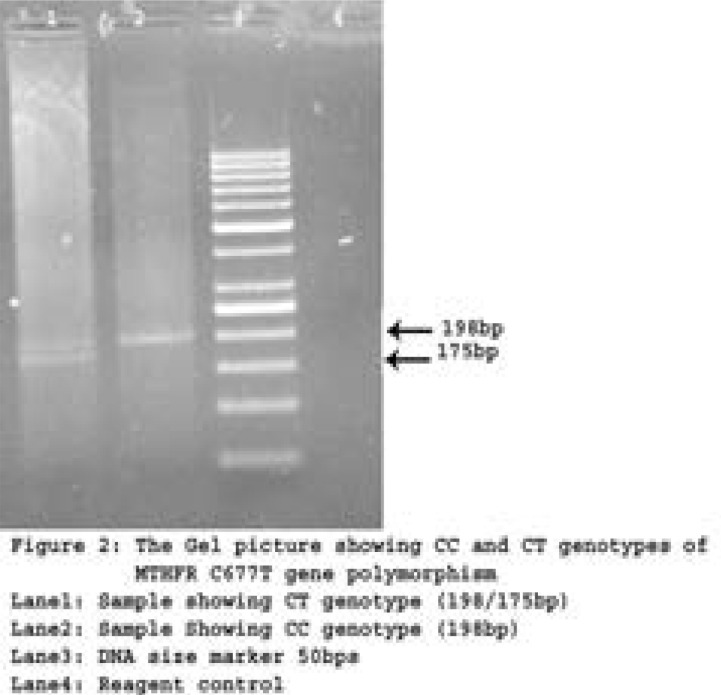
Representative gel picture showing CC & CT genotypes for the MTHFR C677T gene polymorphism

**Figure 2B:**
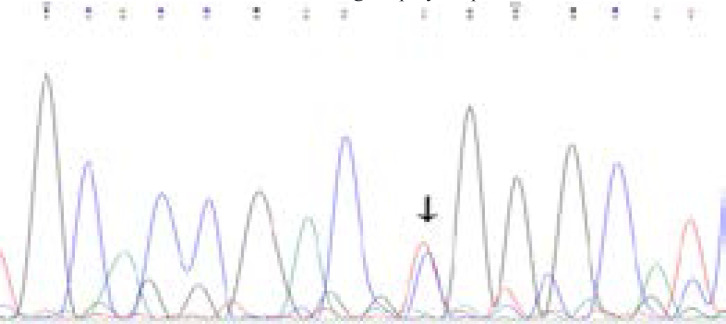
Chromatogram indicating CT (Heterozygous) genotype for the MTHFR C677T gene polymorphism after Sanger sequencing

### Statistical analysis

We used openepi software (Openepi, version 2.3.1, Atlanta, USA) for the statistical analysis. Independent sample t-test analysis was used to analyse the clinical data between KOA cases and controls (Table-2). Clinical data are expressed as mean ± standard deviation (M±SD). Hardy Weinberg equilibrium (HWE) analysis was performed with C677T genotypes of control subjects as described by Khan et al^21^. Genotype and allele frequency (Table-3) were calculated between KOA cases and controls using chi square test. Odds ratios (ORs) and 95% confidence intervals are calculated to estimate the association of genotypes by logistic regression analysis. All the p values less than 0.05 (p<0.05) was considered as statistically significant and association between cases and controls^19^.

**Table 2:** Demographic and clinical findings of the study group

Individual Characteristics	Cases N=200 (Mean±SD)	Controls N=200 (Mean±SD)	P Value
Age	44.04±6.77	43.03±6.09	0.11
**Gender**			
a. Females	119 (59.5%)	114 (57%)	NA
b. Males	81 (40.5%)	86 (43%)	NA
Height	155.135±4.53	155.59±3.9	0.28
**Weight**	**73.275±9.61**	**61.815±7.5**	**0.0001**
**BMI**	**30.44±3.8**	**25.5±3.29**	**0.0001**
Average age of onset	41.125±6.28	NA	NA
**KL Grades**			
Grade 2	120 (60%)	NA	NA
Grade 3	80 (40%)	NA	NA
**Co-morbidities**			
**Hypertensive** **a.– Present**	**91 (45.5%)**	**34 (17%)**	
**b.-Absent**	**109 (54.5%)**	**166 (83%)**	**0.0001**
**Type 2 Diabetes** **a.– Present**	**60 (30%)**	**26 (13%)**	
**b.-Absent**	**140 (70%)**	**174 (87%)**	**0.0001**
Thyroid Dysfunction a.– Present	51 (25.5%)	28 (14%)	
b. -Absent	149 (74.5%)	172 (86%)	0.153
Family History of Osteoarthritis a.– Present	57 (28.5%)	NA	
b.-Absent	143 (71.5%)	NA	

**Table 3:** Distribution of genotypes and allele frequencies of MTHFR (C677T) gene polymorphism on early primary knee Osteoarthritis cases and controls

Genotypes	Cases N=200 (%)	Controls N=200 (%)	Chi square	P Value	OR (95% CI)
CC	176 (88%)	181 (90%)			
CT	24 (12%)	19 (10%)	0.65	0.4206	OR=0.769 95% CI (0.407–1.455)
TT	0	0			
CT vs CC+TT	24 vs 177	19 vs 182		0.4207	OR: 1.2988 95% CI (0.687–2.454)
CT+TT vs CC	25 vs 176	20 vs 181		0.4298	OR: 1.2855 95% CI (0.689–2.398)
**Allele Frequency**					
C	376 (0.94)	381 (0.953)			
T	24 (0.06)	19 (0.047)	0.61	0.434	OR: 1.2800 95% CI (0.689–2.375)

## Results

### Baseline and Anthropometric details of study group

Baseline characteristics and anthropometric details of both KOA cases and controls were provided in Table 2. The mean age ±standard deviation (SD) of cases was 44.04±06.77yrs, and in control group 43.03±06.09yrs. The percentage of females and males were 59.5%, 40.5% respectively in cases whereas in controls 57% & 43%. The ratio of females was higher than males. The mean age of onset ±standard deviation of cases was 41.12 ± 6.28 years. In cases 60% of them were of KL grade 2 and the remaining 40% were KL grade^3^. The percentage of cases with Hypertension, Type 2 Diabetes, Thyroid dysfunction were 45.5%, 30%, 25.5% and in controls 17%, 13%, 14% respectively. Cases with positive family history accounts for 28.5%, indicating genetic predisposition of the disease. Anthropometric measurements like weight, BMI, hypertension, type 2 Diabetes were strongly associated in early primary KOA subjects compared to controls [p=.0001] (Table-2).

### Genotype of C677T -MTHFR gene analysis in cases and controls

The HWE analysis revealed that the genotype frequencies of C677T polymorphism in the control subjects were in the agreement (χ2=0.49; p=0.48). In the current study the genotypes of cases and controls were evaluated for C677T, MTHFR gene polymorphism. The study deviates from the Hardy Weinberg Equilibrium with the complete absence of variant TT genotype in both cases and controls. The percentage of CC, CT and TT genotypes in cases and controls were found to be 88%,12%, 0% and 90%, 10%, 0% respectively. The C allele frequency found to be 0.94 and T allele is 0.06 in cases, whereas in controls is 0.953 and 0.047 respectively. There is no significant difference between genotypes of cases and controls. Dominant, recessive and co-dominant models could not show any statistical significance [OR=1.2855, 95% CI (0.6891–2.3981) p=0.429] (Table 3). MTHFR C677T gene polymorphism lacks the association with early primary knee osteoarthritis.

### Genotype analysis based on gender in KOA cases

Cases were stratified based on the gender and genotype. The percentage of CC genotype in KOA females and males found to be 59.6% and 40.4% respectively. Whereas the percentage of CT genotype in females and males found to be 58% and 42% indicating that there is no significant difference based on gender.

### Genotype analysis based on age in KOA cases

The percentage of CC and CT genotypes in < 40 years age group was 91.5% and 8.5% where as in > 40 years age group it was 86.5% and 13.5%. There is a slight increase in the percentage of CT genotype in >40years age group however; it is not statically significant.

### Genotype analysis based on age and gender in KOA cases

The percentage of CC genotype in <40 years females was 97% where as in males 85%. The percentage of CC genotype in > 40 years females was 85% where as in males 89%. The percentage of CT genotype in females of > 40 years (15%) was higher compared to < 40 years females (3%) and chi square test showed significant difference between females of below and above 40 years of age [chi square=8.79; p=0.003]. However, in males the CT genotype was almost similar in both the age groups and not significantly different.

## Discussion

Osteoarthritis is one of the common musculoskeletal diseases, creating major burden influencing life style in middle and elderly population. The molecular genetics research of OA has been substantially bolstered in the recent years and advancement of the powerful genome wide scans that have revealed a larger number of novel risk loci associated with the disease^22^. Furthermore, the knowledge of candidate gene association studies reveals an avenue for genetic predisposition of the disease. KOA is most prevalent type of arthritis and genetic factors are associated with the occurrence and development of OA 23, 24. In the last decades, many researchers tried to identify the causation variants through GWAS, Meta-analysis, and candidate gene analysis and there by understanding more about the insights of the disease with potential implications to predict predisposition of disease by identifying risk alleles. This in turn paves way to development of novel treatment and management of the disease.

MTHFR gene is considered to be one of the candidate genes for the knee osteoarthritis. The enzyme MTHFR inhibition is responsible for the increase in homocysteine levels. From the earlier studies it was evident that the plasma homocysteine levels increase in individuals with arthritis^25^. Hence, the current study was planned to analyse MTHFR gene polymorphism in south Indian population to assess its possible role in Osteoarthritis.

In the current study, the findings suggest that the MTHFR (C677T) rs1801133 gene polymorphism lack association with early primary knee Osteoarthritis in the South Indian population; however, inclusion of other ethnic groups and larger sample size are needed to fully analyse the role of these polymorphisms with KOA risk prediction. In this study, gender and age-based stratification of genotypes were also categorized however, we could not find any statistical significance between them. But, when we had performed chi square based on age and gender, females below and above the age of 40 yrs showed a significant difference indicating early onset of the disease in presence of risk allele than males of same age group.

Weight is considered as one of the robust risk factors for osteoarthritis and in this study also weight and BMI showed a significant difference between cases and controls, hence, the treating clinicians/surgeons should consider weight reduction also in the treatment plans of OA^26^.

Using regression analysis odds ratio was calculated for genotypes and BMI as cofounder. However, it is not statistically significant. [OR=2.178, 95% CI (0.5580–8.5029) p=0.2625]. It was reported that increased blood pressure is associated with low bone mass and also high risk for fractures. In T2DM patients bone quality was also reduced with high chances of risk for fractures. From the literature it was evident that co morbidities like hypertension and T2DM, interact with subchondral bone remodeling and aggravate the severity of osteoarthritis^27^. In the current study: hypertension, T2DM showed a significant association indicating these play an internal role in the disease mechanism.

According to a study conducted on a Turkish population, the C allele of the MTHFR C677T mutation may be associated with an increased risk of developing osteoarthritis 14. There were only a few studies that focused at the role of the MTHFR C677T gene polymorphism in osteoarthritis. Tasbas et al^28^ previously investigated MTHFR gene polymorphism in Rheumatoid arthritis (RA) patients and discovered that the frequency of MTHFR C677T variant was comparable in Turkish RA patients and control group. Another study from the same population investigated the correlation of MTHFR gene mutation in Alopecia Areata and confirmed that this mutation could be associated with an increased risk of Alopecia Areata 29. A meta-analysis of 16 study populations found that the MTHFR gene C677T SNP has a strong potential association with Rheumatoid Arthritis risk30. As there were paucity of studies in different ethnic groups with the MTHFR C677T gene polymorphism and osteoarthritis, this study makes an impact to the existing literature. To the best of our knowledge this is the first study from South India which evaluated the association of MTHFR C677 T gene polymorphism and early primary knee osteoarthritis.

One of the limitations of this study group is opting the control subjects without the radiologic screening. The other limitation of our study is the low sample size and all the cases were from the same ethnic group. Hence, larger study with a greater number of samples from different ethnic groups will help us to understand the influence of this gene polymorphism in predisposing to Osteoarthritis.

## Conclusion

The current study evaluated the association of MTHFR C677T (rs1801133) gene polymorphism with early primary KOA and the results showed a lack of association. Knee Osteoarthritis is multifactorial and also anticipated to be a result of multiple gene involvement and gene-gene interaction. To the best of our knowledge this is the first study from South India which evaluated the association of MTHFR gene polymorphism with early primary knee osteoarthritis.
